# Long-Term *Saccharomyces cerevisiae* Supplementation Enhances Milk Yield and Reproductive Performance in Lactating Dairy Cows on Smallholder Farms

**DOI:** 10.3390/ani16010032

**Published:** 2025-12-22

**Authors:** Naritsara Suayroop, Vilaivan Khanthusaeng, Aree Kraisoon, Thanya Bunma, Juthamas Nabthonglang, Pakpoom Navanukraw, Theerachai Haitook, Anusorn Cherdthong, Chainarong Navanukraw

**Affiliations:** 1 Department of Animal Science, Faculty of Agriculture, Khon Kaen University, Khon Kaen 40002, Thailand; narisu@kku.ac.th (N.S.); thanyab@kkumail.com (T.B.); juthamasn@kkumail.com (J.N.); theeha@kku.ac.th (T.H.); anusornc@kku.ac.th (A.C.); 2 Department of Animal Science, Faculty of Liberal Arts and Science, Roi Et Rajabhat University, Roi Et 45120, Thailand; vilaivan.reru@gmail.com; 3 Faculty of Agriculture and Agricultural Industry, Surindra Rajabhat University, Surin 32000, Thailand; aree.kri@srru.ac.th; 4 Division of Theriogenology, Faculty of Veterinary Medicine, Khon Kaen University, Khon Kaen 40002, Thailand; pakpna@kku.ac.th; 5 Center of Excellence on Agricultural Biotechnology (AG-BIO/MHESI), Bangkok 10900, Thailand

**Keywords:** nutritional strategy, *Saccharomyces cerevisiae*, milk yield, progesterone, somatic cell count

## Abstract

This study examined the effects of long-term *Saccharomyces cerevisiae* supplementation on feed intake, milk production, milk composition, and selected reproductive indicators in lactating dairy cows. Twenty-four multiparous Holstein–Friesian crossbred cows were blocked by parity and randomly allocated to three treatments: a control group without supplementation (CON; n = 7), live yeast supplementation for 60 days (YS-60; n = 10), and live yeast supplementation for 90 days (YS-90; n = 7). Dry matter intake and body weight gain were significantly higher in cows receiving live yeast, with the greatest responses observed in the YS-90 group (*p* < 0.05). Milk yield and energy-corrected milk were increased by supplementation, particularly in YS-90 cows (*p* < 0.01), along with higher milk fat and lactose concentrations. Somatic cell count was consistently lower in YS-90 cows throughout the 14-week experimental period. Body condition score differed among treatments (*p* < 0.01), with higher values observed in yeast-supplemented cows. Feed efficiency did not differ among treatments. Reproductive parameters, including estrus detection and pregnancy rate, were not significantly affected by live yeast supplementation, although plasma progesterone concentration was higher in supplemented cows (*p* < 0.05). Given the limited number of animals per treatment, reproductive outcomes should be interpreted cautiously. Overall, extended live yeast supplementation improved production performance and udder health, while its effects on reproductive performance warrant further investigation.

## Simple Summary

Early lactation is a challenging period for dairy cows, often associated with high nutrient demands and variable productive responses. This study evaluated the effects of long-term supplementation with *Saccharomyces cerevisiae* on feed intake, milk production, and selected reproductive indicators in lactating dairy cows. Cows receiving live yeast for 90 days showed higher dry matter intake, increased milk yield, higher milk fat and lactose concentrations, and lower somatic cell counts compared to unsupplemented cows. Body condition score was also higher in cows supplemented for 90 days. Circulating progesterone concentrations and pregnancy rate did not differ significantly among treatments. These findings indicate that the duration of live yeast supplementation influences production performance and udder health in lactating dairy cows under the condition of this study.

## 1. Introduction

Enhancing both lactational and reproductive efficiency in dairy cows is a cornerstone of sustainable dairy production, as farmers face growing pressure to balance productivity, animal health, and farm profitability [1]. The early stage of lactation represents one of the most critical physiological periods in a cow’s life, characterized by a sharp rise in nutrient and energy demands to support milk synthesis. During this time, many cows experience a negative energy balance that can trigger metabolic stress and systemic inflammation [1,2]. Such conditions may disrupt the hypothalamic–pituitary–ovarian axis, delay the return to normal estrous cycles, and increase the incidence of postpartum disorders such as mastitis or metabolic diseases [2,3,4]. Therefore, developing nutritional strategies that enhance feed efficiency, stabilize energy metabolism, and strengthen immune function is essential to improving both productivity and reproductive performance in lactating dairy cows [4].

Dairy production systems in Thailand are predominantly based on smallholder farms, which differ markedly from intensive commercial operations in terms of diet composition, feeding consistency, and management practices. These systems commonly rely on locally available forage, agricultural by-products, and variable-quality concentrates, resulting in diets that are often high in structural fiber and subject to fluctuations in energy density and nutrient supply throughout lactation. Consequently, cows managed under smallholder conditions frequently experience inconsistent nutrient intake and prolonged negative energy balance, particularly during early and mid-lactation. These challenges may compromise milk production, body condition recovery, and udder health, even when overall dry matter intake appears adequate [3]. Addressing these constraints requires nutritional strategies that are resilient to feed variability and capable of supporting stable production under less controlled conditions. Previous studies have reported that live yeast supplementation can influence rumen fermentation characteristics and host physiological responses under controlled experimental conditions [5,6]. Among nutritional strategies, live yeast supplementation, particularly with *S. cerevisiae*, has demonstrated clear benefits for rumen health and systemic metabolic function [5,6,7]. These reported responses have been attributed to changes in rumen fermentation patterns, nutrient availability, and host immune status in studies where such variables were directly measured. These responses have been associated with improvements in lactational performance in previous studies, although their relevance under variable smallholder conditions requires further evaluation [2,7]. It is important to note that these fermentation-related outcomes are based on evidence from previous investigations and were not directly assessed in the present study. Live yeast, a widely used commercial yeast product, has been reported to modulate ruminal fermentation by stabilizing rumen pH and increasing the abundance of cellulolytic bacteria [7,8,9]. It also enhances the production of volatile fatty acids (VFAs), particularly acetate and butyrate [5,6,10]. These VFAs serve as major precursors for energy and milk fat synthesis, thereby supporting both productivity and energy balance [11,12].

Yeast supplementation may also exert positive effects on reproductive physiology. Improved energy status and reduced systemic inflammation are known to support the restoration of ovarian activity and luteal function in postpartum cows [2,7]. Furthermore, enhanced nutrient absorption and microbial protein synthesis have been linked to better progesterone production and hypothalamic–pituitary–ovarian axis regulation [12,13,14]. These benefits, however, appear to be time-dependent, as full physiological responses such as immune modulation and microbial stabilization require sustained supplementation to manifest [15,16].

Notably, yeast-derived β-glucans and mannans can modulate immune responses by interacting with host immune cells, improving gut integrity, and enhancing mammary gland defenses [17,18]. This immunomodulatory effect is particularly important for managing somatic cell count (SCC), a critical indicator of udder health and subclinical mastitis [19,20]. Reducing SCC has important implications not only for milk quality but also for overall productivity and reproductive performance, as chronic inflammation is known to impair fertility [21,22].

While the benefits of live yeast supplementation have been documented in both short- and longer-term studies, uncertainty remains regarding the optimal duration of supplementation required to achieve stable and consistent production and reproductive responses [21]. Although several studies have evaluated extended supplementation periods, results remain variable, and it is unclear whether longer durations consistently confer additive or plateauing effects [18,21,22]. Given the potential for cumulative physiological adaptation, evaluating time-dependent responses is essential for developing effective feeding programs.

Therefore, the objective of this study was to compare the effects of live yeast supplementation for 60 and 90 days on growth performance, milk production and composition, SCC, and reproductive responses in lactating dairy cows. It was hypothesized that extended live yeast supplementation would result in more pronounced improvements in production performance, body condition, and selected reproductive indicators compared to shorter supplementation periods. Findings from this study may help refine yeast supplementation strategies with respect to supplementation duration under practical dairy production conditions.

## 2. Materials and Methods

### 2.1. Animals and Experimental Design

All procedures were conducted in accordance with ethical standards and were approved by the Animal Ethics Committee of Khon Kaen University (IACUC-KKU-26/65). The study was carried out on dairy farms located in Ban-Kho Subdistrict, Mueang District, Khon Kaen Province, Thailand. The experiment was conducted across three independent smallholder dairy farms.

The experiment was conducted on three smallholder farms, all of which are members of Khon Kaen Dairy Cooperatives, situated within a 3 km radius. All farms operated under similar housing and management conditions and followed an identical feeding regime based on the same TMR formulation. Cows were housed in free-stall barns with concrete floors and open-sided ventilation, bedded with sand, and milked twice daily in herringbone parlors. All farms used comparable routines for feeding, milking, and reproduction management, as established through cooperative training programs. Routine veterinary services were provided through the cooperative, and herd health records confirmed a low prevalence of mastitis and metabolic disorders during the study period. Prior to the experiment, the participating farmers were well-trained in feeding and milking management. Health monitoring was conducted to ensure uniformity across locations. These management guidelines minimize potential variation due to environmental or management differences, allowing the animals to be treated as a single experimental population. All participating farms belonged to the same cooperative, used a centralized TMR formulation, and followed standardized milking, feeding, and reproductive management protocols. Farm and treatment × farm effects were tested statistically and were not significant (*p* > 0.05) and therefore were excluded from the final model. Preliminary analyses, including farm as a random effect and its interaction with treatment, showed no significant influence on any parameters (*p* > 0.05). Therefore, cows across farms were considered a one-way analysis of variance. Although the enrolled cows ranged from 60 to 150 days in milk (DIM), this interval corresponds to the mid-lactation phase, during which milk yield and intake are relatively stable compared with early lactation. To minimize potential confounding effects of lactation stage, cows were stratified by parity and DIM prior to random allocation, resulting in a balanced DIM distribution across treatments. A total of 24 multiparous Holstein–Friesian × Thai Zebu crossbred cows (n = 24), between 60 and 150 days in milk (DIM), were enrolled in the trial. The sample size of this study (n = 24 lactating dairy cows; 7, 10, and 7 cows per treatment) was determined based on practical and ethical considerations associated with on-farm animal experiments. As described by Charan and Kantharia [23], sample size determination in animal studies is often constrained by animal availability and logistical feasibility. Cows were selected based on parity, days in milk, and milk yield across participating farms, representing the maximum feasible population meeting the inclusion criteria. The possibility of Type II errors was considered when small numbers of cows were assigned to the treatment groups.

Although cows ranged from 60 to 150 DIM, this interval reflects the mid-lactation phase when production traits are relatively stable. To minimize bias, cows were stratified by lactation number and then randomly allocated to treatments, ensuring that DIM distribution was balanced across groups. This sample size reflects the practical herd size constraints of the participating smallholder farms. Because DIM spanned a relatively wide range, cows were first stratified by lactation numbers to balance parity across groups. Within each stratum, animals were then randomly allocated to treatments using a computer-generated random number list, which minimized allocation bias and helped ensure that treatment effects were not confounded by lactation stage. This procedure helped balance DIM distribution across groups and reduced the likelihood that the lactation stage would confound treatment effects. Prior to treatment allocation, baseline parity and days in milk (DIM) were compared among groups to confirm balance. No significant differences were detected among treatments for parity or DIM (*p* > 0.05), confirming that groups were comparable at the start of the experiment. The animals were assigned to one of three treatments under a completely randomized design (CRD): a control group (CON, n = 7); a group supplemented with live yeast for 60 days (YS-60, n = 10); and a group supplemented with live yeast for 90 days (YS-90, n = 7). Cows in the supplemented groups received 10 g/head/day of a commercial live yeast product containing *Saccharomyces cerevisiae* strain CNCM I-1077 (Yea-Sacc^®^, Alltech Biotechnology Corporation Ltd., Nicholasville, KY, USA). The product guaranteed a minimum viable count of 4.28 × 10^7^ colony-forming units (CFU)/g. At the applied inclusion rate, this corresponded to an estimated daily intake of approximately 4.28 × 10^8^ CFU per cow, which is within the effective range reported for lactating dairy cows [21]. Although the supplementation rate expressed in grams per day appears lower than some commercial recommendations, the effective dose delivered was based on colony-forming units (CFU), which is the biologically relevant metric for live yeast supplementation. Live yeast was top-dressed onto the total mixed ration (TMR) and administered once daily during feeding (Table 1).

The cows had an average body weight of 416.28 ± 5.95 kg. Prior to the experiment, all animals were evaluated for general health, reproductive condition, and udder status. Somatic cell count was measured during pre-trial screening, and only cows with SCC below 200,000 cells/mL were selected for inclusion, indicating the absence of subclinical mastitis [24,25]. Cows showing any clinical signs of mastitis—such as udder swelling, redness, abnormal milk secretions, or systemic symptoms like fever were excluded [25,26]. All cows underwent reproductive examinations, including transrectal ultrasonography to assess ovarian structures. To ensure optimal health status, cows were treated subcutaneously with IVOMEC^®^ (Boehringer Ingelheim Animal Health USA Inc., Duluth, GA, USA) (containing 1% ivermectin and 10% clorsulon; Merial) at a dosage of 1 mL per 50 kg body weight, three weeks prior to trial initiation. All cows were monitored daily for health status, and individuals exhibiting clinical signs of illness (e.g., lameness, lumpy skin disease, or injury) were excluded from the trial. Body weight and BCS were recorded weekly throughout the experimental period. The BCS was assessed using a 5-point scale, where 1 = emaciated and 5 = obese, following the method of Edmonson et al. [27]. All assessments were conducted by a single trained evaluator who had prior experience with dairy cow BCS assessment. This approach minimized the risk of inter-observer variation and ensured consistency across the 14-week trial. While a weekly assessment may not capture very small fluctuations, it is considered appropriate for monitoring medium-term changes in body condition in lactating cows. Body weight measurements were also used to calculate Daily dry matter intake (DMI) as a percentage of body weight and in grams per kg metabolic body weight (BW^0.75^).

### 2.2. Diet, Feeding Management, and Feed Sample

During the trial, each cow was housed in an individual pen (3 × 4 m) equipped with its own feed trough and water supply. This arrangement allowed animals to move freely while still enabling precise monitoring of individual intake. Feed was offered as a TMR twice daily (05:00 and 16:00 h). Refusals from each pen were collected and weighed daily before the morning feeding, and feed delivery was adjusted to maintain refusals at ~10% of the amount offered to ensure ad libitum intake. Daily dry matter intake for each cow was calculated as the difference between the amount of feed offered and refusals. The TMR was formulated to meet or exceed the nutrient requirements for early- to mid-lactation Holstein–Friesian crossbred dairy cows according to NRC [28]. The same basal TMR was offered across all treatment groups throughout the experimental period, and the only difference was the supplementation duration of the live yeast culture product. The ingredient composition and chemical analysis of the experimental TMR are presented in Table 1. The major energy sources in the diet included cassava chips, ground corn, and wet cassava pulp, while protein was primarily supplied by soybean meals and dried brewer’s grains. Rice straw was used as the main roughage source. Feed was offered as a complete ration, and individual intake was monitored daily. Daily dry matter intake was then calculated. Because the same basal TMR formulation and ingredient sources were used consistently throughout the experimental period, feed sampling during the final seven days was conducted to verify nutrient composition rather than to characterize temporal variation. During the final seven days of the experimental period, feed samples were collected daily from each treatment group. A 500 g subsample was taken each day and stored for chemical analysis. All feed and refusal samples were dried at 60 °C and ground to pass through a 1 mm screen (Cyclotech Mill, Tecator, Lund, Sweden). Samples were analyzed for proximate composition following AOAC [29] standard methods: dry matter (DM; ID 967.03), crude protein (CP; ID 984.13), ether extract (EE; ID 954.02), and ash (ID 942.05). Acid detergent fiber (ADF) was determined using the AOAC method 973.18 and expressed inclusive of residual ash. Neutral detergent fiber (NDF) was analyzed according to the procedure of Van Soest et al. [30], using α-amylase without sodium sulfite, and values were reported inclusive of residual ash.

Non-fiber carbohydrates (NFC) were calculated to estimate the rapidly fermentable carbohydrate fraction using the following equation:

NFC (%) = 100 − (CP + NDF + EE + Ash).

Total digestible nutrients (TDN) were estimated using a component-based predictive equation suitable for concentrate-based and total mixed rations, as recommended by NRC [28]:

TDN (%) = CP + (2.25 × EE) + NFC + (0.93 × NDF).

These equations provide an estimate of dietary energy supply and were used in the absence of in vivo or in vitro digestibility measurements. Accordingly, TDN values should be interpreted as predictive estimates rather than direct measures of digestible energy. These calculations were used to estimate the dietary energy supply available to support milk production and physiological responses to yeast supplementation. Daily feed cost (USD/day) was calculated for each treatment group based on the quantity of total mixed ration consumed (as DM) and the unit cost of each feed ingredient used in the formulation. Ingredient costs (USD/kg DM) were recorded at the time of the experiment and are shown in Table 1. The total daily feed cost per cow was computed by multiplying the individual DMI by the ingredient cost contribution in the diet.

### 2.3. Blood Sampling and Progesterone Analysis

To assess reproductive hormonal profiles, blood samples were collected weekly via coccygeal venipuncture during the estrous cycle, immediately before feeding the cows. Approximately 7 mL of blood was drawn from each cow into four 1.5 mL micro centrifuge tubes and placed on ice to allow clotting. The samples were centrifuged at 2000× *g* for 10 min, and serum was separated using a 1000 µL micropipette. Serum aliquots were labeled and stored at −20 °C until analysis. Progesterone concentrations were determined using an enzyme-linked immunosorbent assay (ELISA) following the protocol of Navanukraw et al. [31]. Because estrous cycles were not synchronized, weekly progesterone concentrations were used to describe general luteal activity patterns rather than to precisely define cycle stage or ovulatory events.

### 2.4. Milk Sampling and Somatic Cell Count

Before the trial began, udder health was screened using the CMT to exclude cows with suspected mastitis, following the method described by Philpot and Nickerson [32]. Biweekly SCC sampling was intended to monitor general udder health trends rather than to detect short-term mastitis events, which would require more frequent sampling. Throughout the experimental period, milk samples were collected biweekly for SCC and milk composition analysis. Teats were cleaned with 70% ethanol and dried with sterile cloths. After discarding the initial streams of foremilk, composite milk samples (100 mL from all four quarters) were collected into sterile plastic bottles without allowing teat-end contact with the container. Samples were stored at 4 °C until laboratory analysis. SCC was determined using a DeLaval Cell Counter (DCC, Tumba, Sweden). Milk composition, including protein, fat, and lactose, was analyzed using a MilkoScan Minor (FOSS, Hillerød, Denmark), which provided values for fat, protein, lactose, solid-not-fat (SNF), and total solids. Daily milk yield was individually recorded for each cow by weighing the milk collected during both morning and afternoon milking. The fat-to-protein ratio was calculated by dividing milk fat percentage by milk protein percentage. Energy-corrected milk (ECM) was calculated using the equation:

ECM (kg/day) = Milk yield × (0.124 × fat% + 0.073 × protein% + 0.256)

as recommended by NRC [28].

### 2.5. Estrus Detection and Artificial Insemination

The joining period extended throughout the 14-week experimental period. All pregnancies resulted from artificial insemination, and no natural mating occurred. Due to limited animal numbers, the conception rate per AI was not statistically evaluated. Estrus detection was performed twice daily at 06:00 and 18:00 h, with each observation lasting 30 min. Behavioral signs of estrus were evaluated based on the criteria described by Diskin and Sreenan [33], with standing estrus—defined as a cow standing immobile while being mounted by a herdmate used as the primary indicator. Cows showing standing estrus were inseminated 12 h later using frozen-thawed semen provided by the Department of Livestock Development, Thailand. All inseminations were performed by experienced veterinarians.

### 2.6. Statistical Analysis

All data were analyzed using SAS statistical software (Version 9.4; SAS Institute Inc., Cary, NC, USA). For data collected repeatedly over time (e.g., weekly milk yield, progesterone concentration), a repeated-measures analysis was performed using the MIXED procedure. The model included treatment, time, and their interaction as fixed effects, and cow within treatment as a random effect. The MIXED procedure was selected because it does not require the assumption of sphericity and allows explicit modeling of within-cow correlation over time. Residuals from all models were examined graphically (normal probability plots and residual vs. fitted plots) to verify assumptions of normality and homoscedasticity. For repeated measures, several covariance structures were evaluated, and the structure with the lowest Akaike information criterion (AIC) was selected to account for within-animal autocorrelation over time.

Heat detection and pregnancy rates were analyzed descriptively, and inferential statistics were interpreted cautiously due to the limited number of observations per group. For other parameters (e.g., ECM, feed efficiency, SCC), a one-way ANOVA was applied using the GLM procedure. Somatic cell count data were log_10_-transformed prior to analysis to meet assumptions of normality.

The model used was the following:

Yij = μ + τi + εij

where Yij is the observed value of the dependent variable, μ is the overall mean, τi is the fixed effect of treatment (yeast supplementation), and εij is the random error term. No treatment × farm interaction was observed in this study (*p* > 0.05). Thus, the farm effect was excluded from the statistical model. Treatment means were compared using Tukey’s honestly significant difference adjustment to control type I errors and statistical significance was declared at *p* < 0.05. All results are presented as least square means ± standard error of the mean (SEM). Although the group sizes were slightly unbalanced (n = 7, 10 and 7), the MIXED procedure accounts for unequal sample sizes, and treatment comparisons were reported as least square means ± SEM to ensure valid statistical inference.

## 3. Results

### 3.1. Growth Performance and Nutrient Intake Parameters

Neither the farm nor the treatment × farm interaction was significant; therefore, results are presented pooled across farms. As shown in Table 2, supplementation with live yeast significantly improved final body weight and body weight gain in lactating dairy cows. Because cows were enrolled across a mid-lactation DIM range, differences in body weight gain may partially reflect individual variation in lactation stage in addition to treatment effects. Cows in the YS-90 and YS-60 groups had greater final weights and weight gains compared to the control group (*p* < 0.05), with the highest gain observed in the YS-90 group. Additionally, feed intake and DMI were significantly increased in both supplemented groups (*p* < 0.01), indicating increased feed intake and body weight gain in supplemented cows. Initial body condition score did not differ among treatments (*p* > 0.05). Therefore, treatment effects are presented as changes in body condition score over the experimental period. Body condition score also differed significantly among treatments (*p* < 0.01), with the highest score observed in the YS-90 group, followed by YS-60 and control. Dry matter intake expressed as a percentage of body weight did not differ among treatments, whereas DMI relative to metabolic body weight (g/kg BW^0.75^) was modestly higher in the YS-90 group compared with the control (*p* < 0.05) (Table 2).

### 3.2. Milk Yield and Composition

As shown in Table 3, dietary supplementation with live yeast significantly enhanced milk yield in lactating dairy cows. The greatest average daily milk yield was observed in cows receiving live yeast for 90 days (21.94 kg/day), followed by the 60-day group (20.48 kg/day), and the control group (19.75 kg/day) (*p* < 0.01). Energy-corrected milk was significantly greater in the YS-90 group (20.86 kg/day) compared to the YS-60 (19.20 kg/day) and control (18.18 kg/day) groups (*p* < 0.01), indicating increased milk yield and energy-corrected milk with prolonged supplementation.

Milk fat and lactose concentrations were higher in supplemented cows (*p* < 0.01), whereas milk protein, solids-not-fat, total solids, and fat-to-protein ratio were not affected (*p* > 0.05), indicating that live yeast supplementation selectively influenced specific milk components rather than overall milk composition.

Notably, SCC (log_10_) was significantly reduced in the YS-90 group (*p* < 0.01), indicating lower somatic cell counts in cows receiving 90-day supplementation. Feed efficiency (kg milk/kg DMI) remained statistically similar across treatments, while daily feed cost increased with the duration of yeast supplementation (*p* < 0.01) (Table 3). This observation is presented only as an exploratory comparison, as a full economic analysis was beyond the scope of the present study.

### 3.3. Heat Detection and Pregnancy Rates

**Figure 1:**
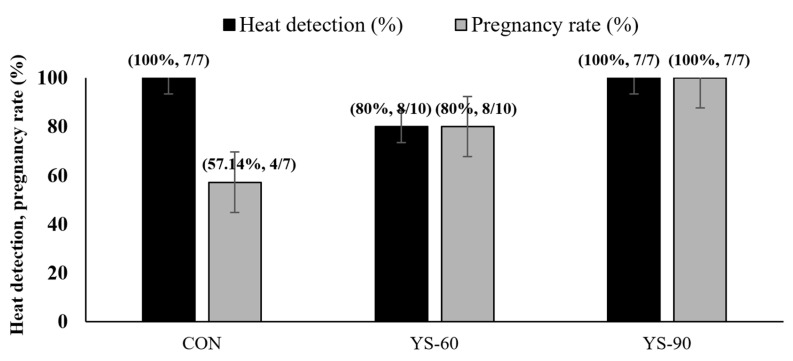
Effect of live yeast supplementation duration on heat detection and pregnancy rates in lactating dairy cows (no supplement (CON), 60-day yeast-fed (YS-60), and 90-day yeast-fed groups (YS-90)).

As illustrated in Figure 1, heat detection rates were high across all groups, with both the control and YS-90 groups achieving 100%, while the YS-60 group showed a slightly lower rate at 80%. In terms of pregnancy outcome, the YS-90 group demonstrated the highest success, reaching 100% (7 out of 7 cows), followed by the YS-60 group at 80% (8 out of 10 cows), and the control group at 57.1% (4 out of 7 cows). Although numerical differences were observed, the Chi-square test indicated no significant effect of treatment on pregnancy rates (*p* > 0.05). The 90-day group showed numerically higher pregnancy rates (100%) compared with the YS-60 (80%) and control (57.1%) groups; however, these differences were not statistically significant (*p* > 0.05) and should be interpreted cautiously. Although numerical differences were observed, reproductive outcomes did not differ statistically among treatments and should be interpreted cautiously due to the limited sample size.

### 3.4. Progesterone Concentration

**Figure 2:**
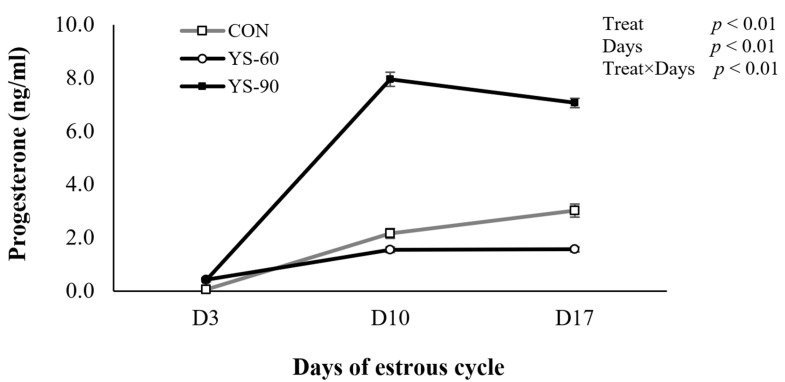
Plasma progesterone profiles during the estrous cycle in cows supplemented with live yeast (no supplement (CON), 60-day yeast-fed (YS-60), and 90-day yeast-fed groups (YS-90)).

A significant treatment effect (*p* < 0.01), day effect (*p* < 0.01), and treatment-by-day interaction (*p* < 0.01) were observed in this study. On day 3, progesterone levels were low and comparable among all groups, indicating a shared baseline early in the cycle. However, by day 10, a clear divergence emerged. Cows in the YS-90 group exhibited a pronounced increase in progesterone concentration, peaking at nearly 8 ng/mL, which was markedly higher than levels observed in the YS-60 and control groups. While progesterone levels in the YS-90 group declined slightly by day 17, they remained elevated relative to the other treatments. In contrast, the YS-60 group maintained relatively stable but lower progesterone levels throughout, and the control group showed only a modest rise. Plasma progesterone concentrations differed among treatments over time; however, substantial individual variation and the limited number of animals warrant cautious interpretation of these patterns (Figure 2).

### 3.5. Somatic Cell Count over Time

**Figure 3:**
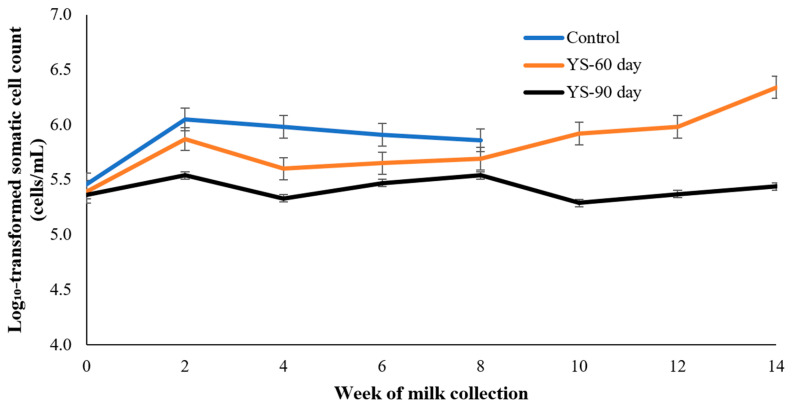
Log_10_-transformed somatic cell count (SCC) dynamics over a 14-week milk collection period in lactating dairy cows receiving no supplementation (CON), live yeast for 60 days (YS-60), or live yeast for 90 days (YS-90). Values are presented as least square means ± SEM. Statistical analyses were performed using log_10_-transformed SCC data to account for the skewed distribution of SCC and to reduce the influence of individual high values under small sample conditions.

Because SCC data are highly skewed and sensitive to individual animal variation, all SCC values were log_10_-transformed prior to statistical analysis and graphical presentation. Figure 3 presents the weekly SCC trends across the 14-week collection period. Cows in the YS-90 group consistently exhibited the lowest SCC values throughout the study. While all groups began at a similar baseline in week 0 (start), the control group showed a progressive increase in SCC, with a pronounced spike occurring after week 10 and peaking at week 14. In contrast, the YS-60 group maintained moderate SCC levels, which fluctuated slightly but remained well below the control group, particularly in the later stages. The YS-90 group demonstrated the most stable profile, with minimal variation and consistently low SCC levels. These results suggest that prolonged yeast supplementation was associated with lower and more stable SCC values over time compared with the control group.

## 4. Discussion

### 4.1. Growth Performance and Nutrient Intake Parameters

Interpretations in this section are based on observed production and physiological responses, while references to rumen fermentation, VFAs, or immune-related mechanisms are discussed only in the context of previous literature and were not directly assessed in the present study. The improvement in final body weight and body weight gain observed in cows supplemented with live yeast, particularly in the YS-90 group, indicates greater body weight gain and body condition recovery in cows receiving prolonged live yeast supplementation. These findings are consistent with previous studies showing that *S. cerevisiae* enhances growth performance by modulating rumen fermentation and increasing the availability of digestible nutrients [6,10,34]. Previous studies have associated live yeast supplementation with changes in rumen fermentation characteristics and fiber utilization [5,35]; however, such mechanisms were not directly evaluated in the present study.

The higher DMI observed in YS-60 and YS-90 groups reflects improved rumen environment and feed palatability, likely due to enhanced microbial activity and reduced subclinical acidosis [36,37]. Furthermore, the increased DMI relative to metabolic body weight in YS-90 cows suggests improved metabolic efficiency, possibly linked to enhanced microbial protein synthesis and energy availability for both lactation and tissue repair [4,12].

The improvement in BCS observed only in cows fed live yeast for 90 days suggests that the duration of supplementation plays a critical role in shifting the cow’s energy balance toward tissue recovery. During early to mid-lactation, cows typically experience a prolonged negative energy balance, where most available energy is directed toward supporting high milk yield [12,20]. While 60 days of yeast supplementation may enhance intake and nutrient digestibility, it might not be long enough to allow for visible changes in body reserves. Ninety days of continuous supplementation was associated with greater body condition recovery compared with shorter supplementation duration [9,12]. Over time, this may have supported greater energy availability for replenishing body fat stores, resulting in a higher BCS [9,27]. This response reflects greater body condition recovery following extended supplementation [1].

These outcomes underscore the value of sustained live yeast supplementation, especially over 90 days, to promote better nutrient utilization, support growth, and maintain energy balance during high metabolic demand phases.

### 4.2. Milk Yield and Composition

The current findings indicate that live yeast supplementation significantly improved milk yield, ECM, milk fat percentage, and lactose concentration, with the most consistent and robust benefits seen in the YS-90 group. The observed increases in milk yield, ECM, milk fat, and lactose in yeast-supplemented cows are consistent with responses reported in previous studies evaluating *S. cerevisiae* supplementation during mid-lactation [35,38,39]. In contrast, milk protein, solids-not-fat, and fat-to-protein ratio were not affected, indicating that the response to live yeast supplementation was component-specific rather than reflective of generalized changes in milk composition. The range of DIM (60–150 days) in the present study represents a stable mid-lactation phase, during which milk fat content is generally less variable compared with early lactation. Consequently, no DIM-related decline in milk fat was detected, and the observed improvements in milk fat percentage with live yeast supplementation can be attributed to treatment effects rather than differences in lactation stage. Although similar production responses have been reported in previous yeast supplementation studies [11,35,38], the present study did not include measurements of rumen fermentation characteristics, microbial populations, or metabolic indicators. Therefore, the observed improvements in milk yield and composition should be interpreted as descriptive production responses rather than attributed to specific underlying mechanisms. Future studies incorporating direct rumen or metabolic measurements are required to elucidate the biological pathways involved.

However, the superior performance in the YS-90 group suggests a time-dependent effect of yeast. The superior performance observed in the YS-90 group indicates a time-dependent response to supplementation, consistent with reports that longer feeding durations are associated with more stable production responses [15]. In contrast, 60 days may not be sufficient to fully establish these changes or achieve maximal lactational benefits.

The lower SCC observed in the YS-90 group indicates reduced somatic cell counts over time compared with the control; however, immune parameters and mastitis culture data were not evaluated, and SCC should therefore be interpreted as a descriptive indicator only [18,40]. The absence of significant effects on milk protein and solids-not-fat content suggests that yeast primarily influences energy-related milk components rather than protein metabolism within this timeframe [10]. The lack of response in milk protein and solids-not-fat content observed in the present study is consistent with reports indicating that the effects of live yeast on milk protein synthesis are variable and often strain-dependent. Several studies have demonstrated that only specific *S. cerevisiae* strains or higher dietary metabolizable protein supply result in measurable increases in milk protein concentration, whereas other strains primarily influence energy-related components such as milk fat and lactose [10,38]. In addition, the cows in this study were fed a standardized total mixed ration based on rice straw as the primary forage source, which provided a relatively consistent and moderate crude protein supply throughout the experimental period. Under such conditions, improvements in rumen fermentation efficiency may preferentially support energy partitioning toward milk fat synthesis rather than altering amino acid availability for milk protein synthesis. Therefore, the absence of changes in milk protein and solids-not-fat likely reflects both strain-specific responses and the characteristics of the basal diet, rather than a lack of biological effect of yeast supplementation.

Although feed efficiency was unchanged, the increased feed cost with longer supplementation must be considered alongside productivity gains and improved udder health. The comparison of feed cost and milk output is presented as an exploratory observation, as a formal economic analysis was beyond the scope of this study [37].

### 4.3. Reproductive Performance

Reproductive variables did not differ statistically among treatments, although numerical differences were observed. Previous studies have shown that even modest improvements in heat detection and conception rates can yield substantial reproductive and economic benefits at the herd level [33]. The YS-90 showed a numerical trend toward the highest heat detection and pregnancy rates, rather than demonstrating the highest, suggesting a possible relationship between supplementation duration and reproductive success [37].

Numerical variation in estrus expression and pregnancy rates was observed among treatments; however, these differences were not statistically significant. Such variation may reflect individual cow differences, stage of lactation, and the limited and unequal sample sizes across groups rather than treatment effects [1,5]. Accordingly, reproductive outcomes should be interpreted cautiously and considered exploratory. Larger, adequately powered studies with balanced group sizes are required to determine whether supplementation duration influences reproductive performance. Another limitation of the present study is the relatively short observation period, which restricted the evaluation of long-term reproductive indicators such as interpregnancy and intercalving intervals. Future studies should extend follow-up duration to assess whether short-term numerical variation in reproductive variables translates into sustained fertility outcomes.

### 4.4. Progesterone Concentration

Progesterone concentrations differed among treatments over time, with substantial inter-animal variability [40]. Progesterone level serves as a key for luteal functions during the estrous cycle. Progesterone production depends on adequate cholesterol availability and luteal steroidogenic activity, both of which are influenced by energy status [2,3]. The observed increase aligns with previous findings that improved nutrient intake enhances corpus luteum development and steroidogenesis [5,12]. Previous studies have reported that yeast supplementation may be associated with increased acetate and butyrate production; however, these rumen fermentation parameters were not measured in the present study and therefore cannot be confirmed [13,14]. In addition, prolonged supplementation may reduce hepatic progesterone clearance by improving metabolic homeostasis and lowering liver blood flow during negative energy balance [22,41].

The relatively lower and stable progesterone levels in the YS-60 group indicate that 60 days may not be adequate for achieving full endocrine recovery or HPO axis stability. The benefits of yeast on immune and reproductive signaling likely require sustained supplementation [15]. Moreover, the lower SCC observed in the YS-90 group supports the anti-inflammatory hypothesis, as systemic inflammation negatively impacts luteal function [21]. Longer supplementation may be necessary to establish optimal progesterone profiles and enhance fertility through both metabolic and immune modulation. No metabolic indicators related to energy balance (e.g., NEFA, BHBA, insulin) were measured in this study; therefore, progesterone results are presented descriptively and should not be interpreted as evidence of improved metabolic status.

### 4.5. Somatic Cell Count over Time

Extended live yeast supplementation was associated with lower and more stable SCC values over the 14-week observation period. The control group experienced a typical post-peak increase in SCC [20], whereas the YS-90 cows maintained low and stable SCC. These results are indicative of improved mammary immune defense and reduced inflammation. SCC is a critical marker of udder inflammation and subclinical mastitis [19]. Because immune indicators and mastitis culture data were not collected, SCC trends should be interpreted as descriptive indicators of udder health rather than evidence of immune modulation. These adaptations develop gradually and require sustained supplementation. Thus, 90 days of yeast feeding allowed full establishment of immune resilience and mammary barrier protection, explaining the consistent SCC suppression compared with the shorter 60-day period. Yeast-derived β-glucans and mannans modulate immune receptors, reduce oxidative stress, and strengthen gut and mammary barrier function [17,22]. These immune benefits are cumulative and require sustained yeast exposure, as previous studies suggest that full systemic immune adaptation may take more than eight weeks [15,16].

The YS-60 group exhibited modest improvement but failed to maintain the consistent SCC suppression observed in the YS-90 group. This highlights the importance of supplementation duration in establishing long-term immune and microbial resilience [42,43]. Furthermore, the lack of change in protein and solids-not-fat content supports the conclusion that SCC reduction was due to improved mammary function rather than milk dilution [10,12]. Collectively, these findings reinforce that extended yeast supplementation—specifically 90 days—is necessary to support udder health throughout lactation and reduce the risk of subclinical mastitis.

We also tested the farm statistically as a fixed effect but found no significant influence or any treatment × farm interaction. This supports the conclusion that environmental variation was negligible under the standardized protocols applied across farms.

## 5. Conclusions

Long-term supplementation with Yea-Sacc^®^ for 90 days improved nutrient intake, body weight gain, milk yield, and milk fat and lactose concentrations, while consistently reducing somatic cell count in lactating dairy cows. These results demonstrate that supplementation duration is a key determinant of response, with the 90-day regimen producing more stable and pronounced benefits than 60 days of supplementation. Reproductive variables, including progesterone concentration and pregnancy rate, were not significantly affected by live yeast supplementation. Although numerical differences and altered progesterone profiles were observed, these outcomes should be interpreted as exploratory and not as confirmed reproductive responses. The findings should be interpreted in light of important limitations, including the small sample size, the wide range of days in milk at enrollment, and the absence of direct measurements of rumen fermentation and immune function. Within these constraints, the present study provides evidence that extended live yeast supplementation enhances productive performance and udder health, supporting the importance of evaluating supplementation duration in dairy nutrition strategies. Further studies with larger populations and integrated rumen and metabolic measurements are required to clarify potential links with reproductive physiology.

## Figures and Tables

**Table 1:** Ingredients and chemical composition of the total mixed ration.

Item	Dry Matter (kg)
Chopped rice straw	19.50
Cassava chip	35.30
Wet cassava pulp	5.00
Dried brewer’s grain	10.00
Soybean meal (44% CP)	17.00
Ground corn	11.00
Urea	1.50
Premix ^1^	0.50
Sulfur powder	0.10
Sodium bicarbonate	0.10
Total	100.00
Cost (USD/kg)	0.27
Chemical composition	
Dry matter (DM, %)	74.50
	% Dry matter
Organic matter	88.12
Crude protein	16.94
Ether extract	4.34
Neutral detergent fiber	24.70
Acid detergent fiber	16.20
Non-fiber carbohydrate	42.14
Total digestible nutrient	79.10

^1^ Premixed in 1 kg consisted of vitamin A 4,000,000 IU, vitamin D3 400,000 IU, vitamin E 4000 IU, vitamin B12 0.002 g, Mn 16 g, Fe 24 g, Zn 24 g, Cu 2 g, Se 0.05 g and I 0.5 g.

**Table 2:** Influence of live yeast feeding duration on growth performance and nutrient intake parameters in dairy cattle.

Item	Live Yeast Supplement			SEM	*p* Value
	CON	YS-60	YS-90		
Number of cows	7	10	7		
Initial body weight (kg)	412.57	416.40	419.86	5.95	0.7206
Final body weight (kg)	416.43 ^b^	432.21 ^a^	448.57 ^a^	3.70	0.0001
Body weight gain (kg)	3.86 ^c^	16.02 ^b^	28.71 ^a^	4.53	0.0018
Body condition score	2.49 ^b^	2.60 ^b^	2.75 ^a^	0.04	0.0001
Dry matter intake (kg/day)	8.58 ^c^	8.83 ^b^	9.03 ^a^	0.09	0.0001
Dry matter intake (%BW)	2.08	2.12	2.15	0.03	0.2586
Dry matter intake (g/kg BW^0.75^)	93.72 ^b^	95.92 ^ab^	98.06 ^a^	0.50	0.0436

^a,b,c^ Mean within a row with different superscripts differ significantly (*p* < 0.05); CON = no supplement; YS-60 = Yeast supplement 60 days; YS-90 = Yeast supplement 90 days; SEM = Standard error of mean.

**Table 3:** Effect of *S. cerevisiae* supplementation on milk production and composition.

Item	Live Yeast Supplement			SEM	*p* Value
	CON	YS-60	YS-90		
Milk yield (kg/day)	19.75 ^c^	20.48 ^b^	21.94 ^a^	0.35	0.0001
Milk composition, %					
Fat	3.51 ^b^	3.63 ^a^	3.73 ^a^	0.04	0.0001
Protein	3.14	3.17	3.18	0.12	0.1295
Lactose	4.26 ^b^	4.71 ^a^	4.76 ^a^	0.06	0.0003
Solid not fat	8.63	8.64	8.58	0.12	0.3261
Total solid	12.15	12.27	12.31	0.35	0.2518
Fat: protein	1.12	1.15	1.17	0.03	0.5243
Log_10_ somatic cell count	1.80 ^a^	1.85 ^a^	1.42 ^b^	0.07	0.0024
Feed efficiency					
kg milk/kg feed DM	2.31	2.30	2.35	0.03	0.8320
ECM (kg/day)	18.18 ^c^	19.20 ^b^	20.86 ^a^	0.35	0.0001
Cost (USD/day)	8.92 ^c^	9.35 ^b^	9.88 ^a^	0.02	0.0001

^a,b,c^ Means within a row with different superscripts differ significantly (*p* < 0.05) (CON = no supplement; YS-60 = Yeast supplement 60 days; YS-90 = Yeast supplement 90 days; SEM = Standard error of mean; ECM = Energy corrected milk; ECM corrected for 4% fat and 3.4% protein according to the equation Milk yield × (0.124 × fat% + 0.073 × protein% + 0.256), as recommended by NRC [28].

## Data Availability

The original contributions presented in this study are included in the article. Further inquiries can be directed to the corresponding authors.
